# Synergistic Effects of the Si/Al Stoichiometry and Catalyst Content on the Growth Mechanism of Mullite Whiskers

**DOI:** 10.3390/ma19102065

**Published:** 2026-05-15

**Authors:** Haihong Zhang, Fangli Yu, Haifu Li, Haibo Li, Qiang Zhi, Bin Li, Fengli He, Yeye Liu

**Affiliations:** 1 College of Materials Engineering, Xihang University, No. 259, West Second Ring Road, Xi’an 710077, China; 201607046@xaau.edu.cn (F.Y.); 201904011@xaau.edu.cn (F.H.);; 2 Shaanxi Haichuang Industrial Development Co., Ltd., No. 415, Taihua North Road, Xi’an 710016, China

**Keywords:** mullite whiskers, fluorine catalysis, sol–gel method, Si:Al ratio, growth mechanism

## Abstract

**Highlights:**

**What are the main findings?**

- Reveals the decisive influence of the Al_2_O_3_/SiO_2_ molar ratio and its interaction with HF on whisker morphology.
- Identifies two distinct catalytic reaction pathways for mullite whisker formation under fluorine catalysis.
- Elucidates the correlation mechanism between the catalytic pathway and phase transformation temperature in the Al–Si–F system.

**What are the implications of the main findings?**

- Deepens the understanding of the formation mechanism of mullite whiskers prepared via fluorine catalysis.
- Provides a theoretical basis for tailoring whisker morphology and controlling the corundum phase transformation.

**Abstract:**

In this study, mullite single-crystal whiskers were prepared by sintering mullite gel powders using HF as a catalyst via the sol–gel process. The effects of the Al_2_O_3_:SiO_2_ molar ratio on the morphology of mullite whiskers in the Al–Si–F system were comprehensively explored during the catalytic reaction. Furthermore, the synergistic effects of the Si:Al ratio and the catalyst content on the growth mechanism of mullite whiskers were evaluated. The morphological characteristics of the whiskers were determined using transmission electron microscopy and scanning electron microscopy. Moreover, morphological parameters, including the diameter and length of whiskers, were statistically analyzed using the Image J software. Additionally, the compositional variation and phase evolution during the whisker growth process were examined via energy-dispersive spectroscopy and X-ray diffraction, respectively, and the corresponding growth mechanism was elucidated. When HF-mediated catalysis reaches a sufficient level (Al_2_O_3_:SiO_2_:HF = 1:1.5:4.3), the low SiO_2_ content in the system leads to Al enrichment and the formation of flake-shaped Al_2_O_3_ structures, indicating an effect analogous to that of increasing catalyst content. Conversely, the simultaneous reduction in the contents of HF and SiO_2_ induces different catalytic reactions because of their synergy. Specifically, at relatively low SiO_2_ and HF contents, F ions enter the Al–Si–O system via SiF_4_, leading to the generation of fluorine-containing topaz, which subsequently transforms into mullite. At relatively high SiO_2_ and HF contents, mullite can be directly synthesized via the reaction of AlF3 and SiF_4_. With a gradual reduction in the SiO_2_ and HF contents, the mullite whiskers exhibit a varying morphology, predominantly transitioning from rod-shaped to flake-shaped and subsequently to rod-shaped structures. This is due to the synergistic effects of the phase transformation and catalytic reactions within the Al–Si–O system.

## 1. Introduction

Mullite is the only crystalline aluminosilicate compound in the Al_2_O_3_–SiO_2_ binary system that can maintain stability at high temperatures and normal pressure. Its fundamental structural units are composed of Si–O tetrahedra and Al–O octahedra. In particular, AlO6 octahedra are arranged along the c-axis with common edges, serving as the structural backbone of mullite. These structural characteristics govern the anisotropic growth behavior of mullite, and under appropriate crystallization conditions, mullite crystals inherently exhibit a rod-shaped or needle-shaped whisker morphology along the c-axis [1]. Owing to the extremely small diameter of mullite whiskers, the incorporation of conventional defects within the crystal material is difficult. This results in a highly ordered atomic arrangement and a near-ideal crystal structure, and consequently, the mechanical performance is approximately equivalent to the corresponding theoretical predictions [2]. Compared with other morphologies, such as particles or fibers, whiskers offer a unique combination of a high aspect ratio and near-perfect crystalline structure, enabling more effective load transfer and crack deflection in composite matrices [3]. In addition to excellent mechanical properties, mullite whiskers exhibit exceptional comprehensive properties, such as high thermal stability, creep resistance, low thermal expansion coefficient, low electrical conductivity, and high corrosion resistance. Furthermore, the raw materials required for the synthesis of mullite (aluminosilicates) are easily available. These characteristics thus make mullite whiskers irreplaceable as reinforcing and toughening agents in ceramic, metal, and polymer composites, making them highly attractive for applications that demand both high-temperature durability and mechanical robustness, including high-temperature filters, thermal insulation materials, and structural ceramics [4].

Recently, mullite whiskers have been synthesized via numerous approaches, including the most commonly used methods such as powder calcination, mineral decomposition, sol–gel processing, molten-salt synthesis, and in situ growth [5]. These synthetic strategies correspond to two growth mechanisms, namely, vapor–solid (V–S) and liquid–solid (L–S) approaches. Therefore, they can be classified into two main categories, i.e., solid-phase catalytic method and molten-salt medium method. The molten-salt medium method provides a molten-salt liquid-phase environment for the catalytic reaction, and the products are generated via an L–S reaction. Notably, although this method facilitates a reduction in the reaction temperature, it commonly compromises the purity of the final product. The solid-phase catalytic method involves the calcination of catalyst-containing raw material powders, resulting in a V–S reaction during high-temperature sintering to form whiskers. In the widely used powder calcination and mineral decomposition methods, sintered powders are produced via the solid-phase route, whereas the sol–gel method employs the wet-chemical pathway to generate sintered powders. Furthermore, the most extensively used catalysts are those containing fluorine, as studies have demonstrated that fluorine-containing additives, such as AlF_3_, can effectively promote the formation of mullite whiskers with high purity [6,7].

Various distinct perspectives have been proposed for the growth mechanism and the reaction process of mullite whiskers in the presence of fluorine-containing catalysts. Currently, two prevalent perspectives have been identified: (1) the fluorine-containing topaz phase is an important intermediate product, and the reaction proceeds via the initial formation of the topaz phase and the subsequent transformation into mullite [6,8,9]; (2) the catalytic reaction does not involve fluorine-containing aluminosilicate salts, and mullite is directly generated from various fluorides [10,11,12]. However, the two aforementioned theories exhibit a lack of consonance. Based on the in-depth understanding of the catalytic mechanism of fluorine catalysts and the effects of the catalyst content on whisker growth, liquid hydrofluoric acid is employed as the catalyst in this study to prepare high-purity sintered powders via the sol–gel method. Furthermore, the effects of the Si:Al ratio and catalyst content on the catalytic reaction of mullite whiskers are systematically investigated, particularly focusing on the synergy between these two influential factors.

## 2. Experimental Procedures

### 2.1. Sample Preparation

Owing to the generation of gaseous intermediate products, fluorine-containing catalysts consume a certain amount of Si during the catalytic process, which leads to aluminum enrichment in the reaction system under high-temperature conditions [13]. Therefore, several studies have prepared mullite by employing silicon-rich compositions featuring stoichiometric ratios higher than the normal ratio (3:2) of mullite [14]. This study also focuses on the complex interaction between the Si:Al ratio and catalyst content in a silicon-rich system. Accordingly, the molar ratios of the effective oxides, Al_2_O_3_ and SiO_2_, were set to 1:1.5, 1:1.25, and 1:1 to investigate the effects of different Si:Al ratios on the catalytic process and performance. When examining the exclusive effects of the Si:Al ratio, the catalyst content was maintained constant. Based on previous experimental results [13], the Al_2_O_3_:HF molar ratio was set as 1:4.2 to ensure the complete catalytic conversion of the silicon-rich reactant. The synergistic effects of the Si:Al ratio and catalyst content were determined by simultaneously varying these two factors while maintaining a constant SiO_2_:HF molar ratio of 1.5:4.2. Accordingly, the HF molar ratios corresponding to the SiO_2_ molar ratios of 1.5, 1.25, and 1 were 4.2, 3.5, and 2.8, respectively. The specific experimental groups and compositions used in this study are listed in Table 1.

Mullite whiskers were prepared using polymeric aluminum chloride sol as the aluminum source, tetraethyl orthosilicate (TEOS) as the silicon source, HF as the catalyst, and anhydrous ethanol as the dispersion medium. Polymeric aluminum chloride was supplied by Suzhou Yongda Fine Chemical Co., Ltd. (Suzhou, China), and all other chemical reagents were procured from Sinopharm Chemical Reagent Co., Ltd. (Shanghai, China). The polymeric aluminum chloride sol and TEOS were weighed based on the various molar ratios of the effective oxides, Al_2_O_3_ and SiO_2_. Thereafter, they were added to anhydrous ethanol and magnetically stirred at ambient temperature for 1 h to produce a mixed sol. A certain amount of HF was added to the as-obtained mixed sol and stirred for 2 h to obtain the precursor sol. An ammonia solution was added dropwise to the as-prepared precursor sol to form a gel. The gel powders were thoroughly dried, precalcined at 500 °C, and ground using a mortar to obtain uniform powders. Subsequently, the as-formed powders were calcined in a sealed corundum crucible at 1500 °C for 1 h to yield mullite whiskers.

### 2.2. Microstructure Characterizations

The microstructure characteristics and elemental compositions of the as-prepared samples were examined using a JEOL JSM-6510A (Tokyo, Japan) scanning electron microscopy instrument equipped with an energy-dispersive X-ray spectroscopy (EDS) system. Based on the SEM images, the morphological parameters, such as the diameter and length, of mullite whiskers were statistically analyzed using the Image J software (1.54P).

Furthermore, the whiskers were ground into powders for X-ray diffraction (XRD) analysis using a D/max-3c diffractometer (Bruker D8 Advance, Karlsruhe, Germany) at 40 kV and 100 mA utilizing Cu Ka radiation. The as-obtained data were analyzed using the High Score Plus software (3.0 e). Semiquantitative calculations were conducted based on reference intensity ratio (RIR) values. The lattice parameters and growth directions of the single-crystal whiskers were analyzed using a JEOL JEM-2100F (Tokyo, Japan) high-resolution field-emission transmission electron microscopy (HRTEM) equipment.

## 3. Results and Discussion

### 3.1. TEM Analysis of Whiskers

**Figure 1:**
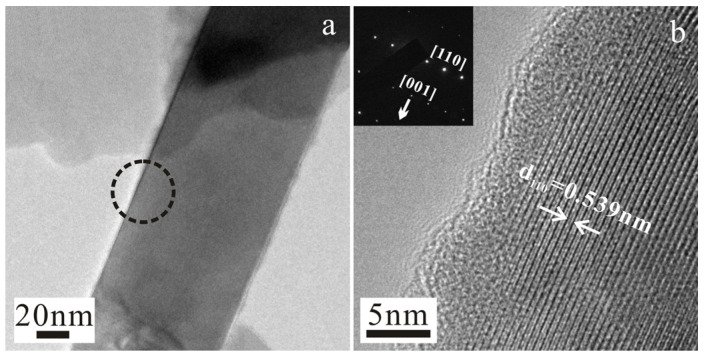
HRTEM images and SAED pattern of the mullite whisker: (**a**) low-magnification TEM image. (**b**) Enlarged image of the region indicated by the dashed line in (**a**); the inset presents the SAED pattern.

Based on conclusions from our previous study and the experimental results discussed below, the Al_2_O_3_:SiO_2_:HF molar ratio of 1:1.5:4.2 yields mullite whiskers with the most complete catalysis and the most pronounced aspect ratio. Therefore, whiskers prepared using this specific composition were considered the base group. After dispersion, the whiskers were collected on copper grids for TEM analysis. Figure 1 depicts the TEM images of a single ultrathin whisker. From Figure 1a, the whisker has a prominent aspect ratio and features a uniform diameter along its entire length. Moreover, the whisker surface is coated with a thin amorphous layer. The HRTEM image in Figure 1b confirms the single-crystalline characteristics of the whisker. The lattice fringes of the densest crystal planes exhibit an interplanar spacing of 0.539 nm, which is consistent with the maximum lattice spacing, d, of the (110) crystal plane of the mullite phase (PDF01-088-07826). The nearest adjacent diffraction spot relative to the central spot in the corresponding selected area electron diffraction (SAED) pattern represents the (110) crystal plane of the mullite phase, whereas another adjacent spot that does not lie on the same straight line corresponds to the (001) crystal plane. This indicates that the primary crystal orientation is [001], as indicated by the arrow, and the [110] crystal orientation is perpendicular to [001] on the same crystal plane [15]. The most densely packed (110) crystal plane is perpendicular to the plane of the HRTEM image. The arrow in Figure 1b indicates the growth direction of the whisker, and the corresponding SAED pattern indicates that the growth direction of the mullite whisker is along the c-axis. The aforementioned crystallographic characteristics are consistent with the prismatic crystal structure of natural mullite crystals. Therefore, the TEM analysis confirms that the as-prepared sample is composed of a single mullite crystal.

### 3.2. Effects of the Si:Al Ratio on the Morphology of Mullite Whiskers

**Figure 2:**
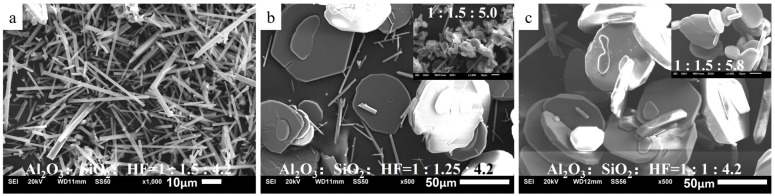
SEM images of the mullite whiskers obtained with decreasing SiO_2_ content: (**a**) base group featuring the Al_2_O_3_:SiO_2_:HF molar ratio of 1:1.5:4.2; (**b**,**c**) groups in which the SiO_2_ proportion is reduced to 1.25 and 1, respectively, while maintaining the proportions of the other components the same as those of the base group. The reference images in the top-right corners of (**b**,**c**) show the whisker morphology obtained with increasing HF content at a fixed Al_2_O_3_:SiO_2_ molar ratio of 1:1.5, with the labels denoting the corresponding Al_2_O_3_:SiO_2_:HF molar ratios.

To reveal the effects of different Al_2_O_3_:SiO_2_ ratios on the morphology of the mullite whisker, the SiO_2_ proportion of the base group was reduced from 1.5 to 1.25 and 1 while maintaining the proportions of the other two components invariant. Figure 2 illustrates the SEM images of the as-prepared mullite whiskers. The mullite whiskers of the base group exhibit a well-defined morphology, demonstrating a uniform diameter and a prominent aspect ratio, as shown in Figure 2a. Furthermore, no clusters of short whiskers are observed, indicating that the mullite whiskers are in the stage of complete growth rather than the initial nucleation phase. When the Al_2_O_3_:SiO_2_:HF ratio is 1:1.5:4.2, the catalyst content is sufficient to achieve effective catalysis. However, Figure 2b,c exhibit a hexagonal or nearly circular plate-shaped morphology. As depicted in Figure 2b, only a few rod-shaped whiskers are noted, whereas a flake-shaped morphology is observed throughout the image in Figure 2c. Based on our previous study, the appearance of the flake-shaped structure, which is composed of Al_2_O_3_, typically indicates excessive catalysis [16].

**Figure 3:**
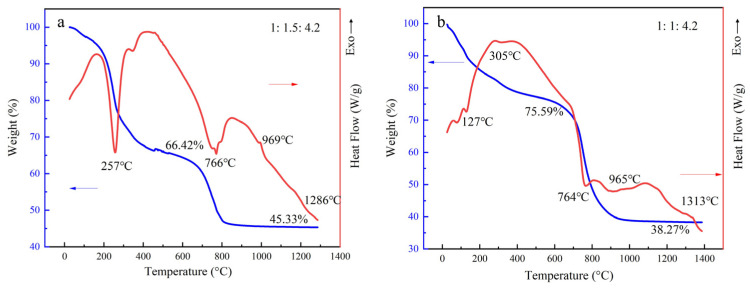
DSC/TG curves of the mullite whiskers: (**a**) Al_2_O_3_:SiO_2_:HF molar ratio of 1:1.5:4.2; (**b**) Al_2_O_3_:SiO_2_:HF molar ratio of 1:1:4.2.

The DSC-TG data presented in Figure 3 provide substantial evidence for the occurrence of excessive catalysis. The endothermic peak observed at approximately 760 °C corresponds to the decomposition of fluorides [17], which leads to a mass loss in the system and also marks the temperature at which the catalytic reaction occurs vigorously. A comparison of the TG curves at this temperature between Figure 3a,b reveals that for the 1:1 ratio sample in Figure 3b, the mass of the system decreases from 75.59% to 38.27%, representing a mass loss significantly greater than that observed for the 1:1.5 sample in Figure 3a. This finding demonstrates that the flake-shaped sample underwent a more intensive catalytic process compared to the rod-like whiskers.

**Figure 4:**
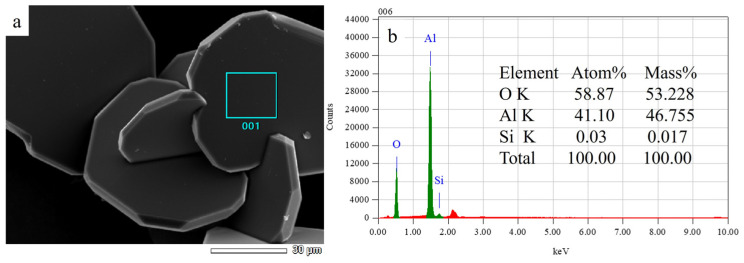
Microstructure of the flake-shaped material: (**a**) SEM image and (**b**) EDS analysis results.

Moreover, the flake-shaped material is analyzed via EDS. As presented in Figure 4, the Si content of the flake-shaped material (the position of the square in the subpanel Figure 4a) is limited, whereas the atomic and mass ratios of the O and Al elements are approximately equivalent to those of Al_2_O_3_ (O:Al = 53:47 (wt.%)), which is consistent with the previous results.

The morphological parameters of the mullite whiskers and flake-shaped Al_2_O_3_ material were statistically analyzed based on the SEM images using Image J software. The corresponding results are summarized in Table 2.

From Table 2, when the Al_2_O_3_:SiO_2_ ratio is changed from 1:1.5 to 1:1.25, the mullite whiskers exhibit a substantial transformation into flake-shaped Al_2_O_3_ structures. When the SiO_2_ proportion is further reduced to 1, the diameter and thickness of the flake-shaped Al_2_O_3_ structure increase. Notably, a comparison with the reference images in the top-left corners of Figure 2b,c reveals that this variation trend aligns with that observed with increasing HF content: at a fixed Al_2_O_3_:SiO_2_ molar ratio of 1:1.5, as the catalyst content increases from 4.2 to 5.0 and 5.8, the whisker morphology similarly evolves from rod-like to small flakes and then to larger, thicker flakes. These findings suggest that a reduction in the SiO_2_ content can change the originally sufficient catalysis into excessive catalysis.

Additionally, in our previous study [13], when HF was used as the catalyst, the initial generation of the gaseous SiF_4_ product was essential for mullite formation. Although SiF_4_ was involved in the subsequent reactions, a certain amount of SiF_4_ might escape from the system, resulting in an aluminum-rich environment. This suggested that during the growth of whiskers in the presence of a catalyst, the fluorine element can induce the excessive consumption of the silicon content in the system. The mass loss observed in the TG curves in Figure 3 also supports this view.

This finding explains why, in the present study, although the Si content in the base group (Al_2_O_3_:SiO_2_ = 1:1.5) was higher than that in the stoichiometric ratio of 3:2 mullite (3Al_2_O_3_·2SiO_2_), it is sufficient to facilitate the complete catalytic formation of whiskers in the presence of the HF catalyst. Based on this ratio, when the Al_2_O_3_ and HF contents remain unchanged, the reduction in the SiO_2_ content leads to an aluminum-rich system, consequently facilitating the generation of numerous flake-shaped Al_2_O_3_ structures.

### 3.3. Synergistic Effects of the Si:Al Ratio and Catalyst Content

During the complex synthesis of mullite whiskers within the fluorine-catalyzed Al–Si–O system, the catalyst influences the phase transformation of the Al–Si–O system. Reducing the SiO_2_ content by adjusting the Al_2_O_3_:SiO_2_ ratio leads to an effect analogous to that of increasing HF content. This analogy suggests that the HF content and the Al_2_O_3_:SiO_2_ ratio interact in influencing the catalytic performance. Furthermore, experiments are conducted by maintaining the SiO_2_:HF molar ratio at 1:2.8 while decreasing the HF and SiO_2_ contents to further investigate the synergistic effects and reaction mechanism, and the obtained results are presented in the subsequent sections.

**Figure 5:**
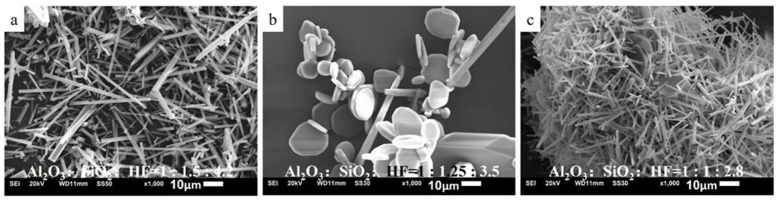
SEM images of the mullite whiskers obtained when the SiO_2_ and HF contents are simultaneously reduced: (**a**) base group featuring an Al_2_O_3_:SiO_2_:HF ratio of 1:1.5:4.2; (**b**,**c**) groups in which the Al_2_O_3_:SiO_2_:HF ratio is decreased to 1:1.25:3.5 and 1:1:2.8, respectively, while maintaining the SiO_2_:HF ratio at 1:2.8.

Figure 5 depicts the SEM images of the mullite whiskers obtained when the SiO_2_ and HF contents are simultaneously decreased. With the simultaneous reduction in the SiO_2_ and HF contents, the sample exhibits mixed morphological features, demonstrating flake-shaped structures and rod-shaped whiskers. From Figure 5b, at the lowest SiO_2_ and HF contents, the proportion of flake-shaped Al_2_O_3_ structures is maximum, whereas that of rod-shaped whiskers exhibits an increasing trend.

**Figure 6:**
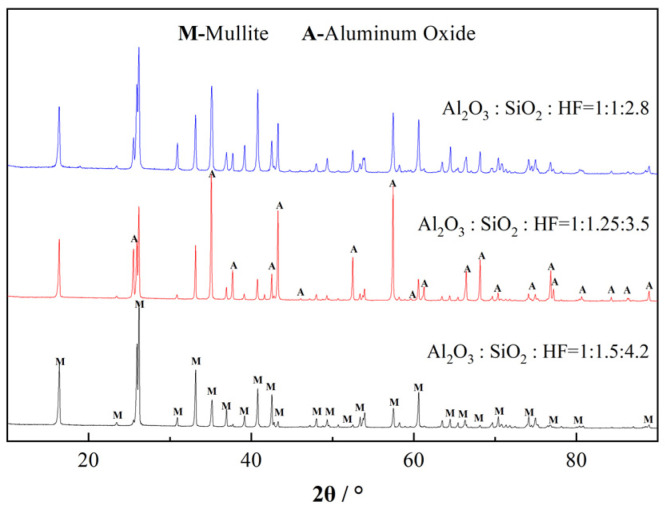
XRD patterns of the whiskers featuring different Al_2_O_3_:SiO_2_:HF ratios.

The phase composition of the samples was determined via XRD analysis. Furthermore, a semiquantitative analysis was conducted. The corresponding results are presented in Figure 6 and Table 3. The phase composition detected via XRD is consistent with the SEM observations. The samples synthesized using the three specified compositional ratios are composed of mullite and alumina phases. However, the base group exhibits the maximum mullite phase content of 88%; therefore, the presence of flake-shaped alumina is negligible, as evidenced in the SEM image. When the SiO_2_ and HF contents are reduced to an intermediate level, alumina is the dominant phase, exhibiting a proportion of >50%, and numerous flake-shaped structures are observed in the SEM image. A further reduction in the SiO_2_ and HF contents increases the mullite phase content to 68%, which is consistent with the predominant rod-shaped structures observed in the SEM image.

**Figure 7:**
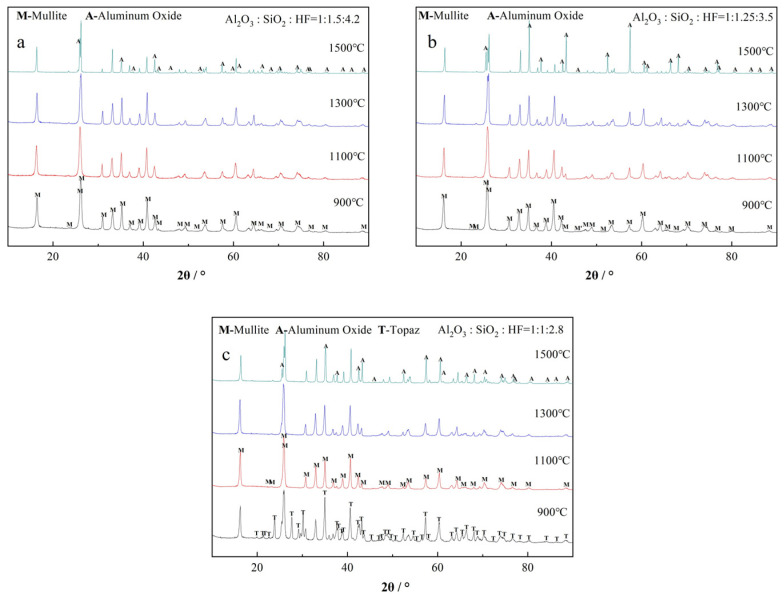
XRD patterns of the as-prepared whiskers featuring various Al_2_O_3_:SiO_2_:HF ratios of (**a**) 1:1.5:4.2, (**b**) 1:1.25:3.5, and (**c**) 1:1:2.8 after sintering at different temperatures.

To clarify the phase transformation process of the samples featuring different compositional ratios, they were analyzed via XRD after sintering at different temperatures. The corresponding results are presented in Figure 7 and Table 4.

The XRD patterns depicted in Figure 7 reveal two distinct trends. First, at high SiO_2_ and HF contents, the formation of the alumina phase begins at 1500 °C; however, the formation temperature decreases with decreasing SiO_2_ and HF contents. At an intermediate ratio, the alumina phase is detected at 1300 °C, and when the Al_2_O_3_:SiO_2_:HF molar ratio is 1:1:2.8, the alumina phase appears at 1100 °C.

Second, at the lowest SiO_2_ and HF contents, the topaz phase (Topaz 01-082-1237) is initially generated at a relatively low temperature, followed by its phase transformation into the mullite phase. Conversely, for the other two compositional ratios, the mullite phase is directly generated without the preliminary formation of the topaz phase. These results reveal that after the simultaneous changes in the SiO_2_ and HF contents, two types of catalytic processes (pathways) concurrently occur because of the synergistic effects. Specifically, when the SiO_2_ and HF contents are low, Si and F ions preferentially generate the high-silicon topaz phase (Al:Si atomic ratio = 2:1), Al_8.0_Si_4.0_O_18.4_F_5.6_H_2.4,_ which gradually transforms into the mullite phase. Conversely, when the SiO_2_ and HF contents are high, a high-alumina mullite phase (Al:Si atomic ratio > 4:1), Al_4.8_Si_1.2_O_9.6_/Al_5.65_Si_0.35_O_9.175_, is preferentially formed.

## 4. Formation Mechanism of Mullite Whiskers

The formation of mullite whiskers is influenced by numerous factors, including complex phase transformation within the Al–Si–O system and the catalytic effects of the F element. These two aspects are discussed in the subsequent sections.

### 4.1. Analysis of the Phase Transformation Mechanism in the Al–Si–O System

Generally, the formation of mullite, which is an intermediate phase within the Al_2_O_3_–SiO_2_ system, depends on the simultaneous diffusion of Al and Si atoms. Compared with that of the alumina phase, the nucleation of the mullite phase is facile; however, its grain growth is more challenging. Studies on the crystallization kinetics of alumina and mullite reveal that when the molecular-level mixing (single-phase colloid) of the Al and Si components in the system is achieved, the onset temperature of mullite crystallization is relatively low (900–1000 °C). Furthermore, the nucleation energy barrier is comparatively low, spanning the range of 290–400 kJ/mol [18]; however, the activation energy required for grain growth is relatively high and is in the range of 900–1400 kJ/mol [19,20]. In contrast, for the dual-phase colloid, the formation of the mullite phase occurs in two stages: the nucleation and growth of an unstable transient mullite phase, followed by a gradual transformation of the 2:1-type aluminum-rich tetragonal mullite into the 3:2-type orthorhombic mullite [21]. The crystallization activation energy of mullite in such colloids is approximately 1000 kJ/mol [16]. Consistent with this two-stage formation process, the sol synthesized in this work exhibits the characteristic transition from an unstable transient mullite phase to the stable 3:2-type orthorhombic mullite, as confirmed by the phase transformation analysis presented in Table 4. As for the 1:1.5:4.2 group in Table 4, where only a single phase (Al_4.8_Si_1.2_O_9.6_ (96-900-1323)) was detected across the entire temperature range, this discrepancy can be explained by the fact that the transformation from the unstable transient mullite phase to the stable phase likely continues even up to 1500 °C. This is because, relative to the stable 3:2-type orthorhombic mullite, Al_4.8_Si_1.2_O_9.6_ (96-900-1323) remains an alumina-rich transitional phase.

Meanwhile, the crystallization kinetics of alumina phases demonstrate that the transformations among metastable alumina phases are characterized by low activation energies, rendering them facile. In contrast, the critical transformation from the metastable alumina phase to the stable α-alumina phase is classified as a lattice-reconstruction-type phase transformation process, which involves the movement of oxygen ions with large ionic radii and thus necessitates a substantial activation energy. During this process, the major portion of the energy is utilized to overcome the nucleation energy barrier for the formation of α-alumina nuclei, whereas only a minor portion of energy is used as the activation energy for the subsequent grain growth [22]. Specifically, the growth kinetic analysis of the α-alumina phase demonstrates that its nucleation activation energy is 850–700 kJ/mol [23], whereas the growth activation energy is 330–640 kJ/mol [21]. Accordingly, in the Al–Si–O system, the nucleation temperature of the mullite phase is lower than that of the alumina phase; therefore, the mullite phase is generated first in the system, followed by the formation of the alumina phase. Research on the high-temperature creep mechanism reveals that the recrystallization of mullite occurs at high temperatures, which promotes the precipitation of alumina particles from the mullite phase [24].

Accordingly, for the dual-phase colloidal Al–Si–O system in this study, an unstable transient mullite phase is initially generated at low temperatures and subsequently undergoes a gradual transformation to form a stable phase, which is followed by recrystallization at high temperatures to yield the alumina phase. Considering the specific experimental data of this study, the phase transformation process of the Al–Si–O system can be described as follows:

Aluminum-rich transient mullite phase (900 °C) → stable orthorhombic mullite phase (900–1500 °C) → recrystallization and precipitation of the alumina phase (1100–1500 °C).

During this process, the reduction in the Si content facilitates the formation of an aluminum-rich environment. As listed in Table 4, the M’ phase (Al_5.65_Si_0.35_O_9.175_) formed at the intermediate ratio contains a higher Al content than the M phase (Al_4.8_Si_1.2_O_9.6_). In this crystal structure, the concentration of Al ions in the matrix and the mullite lattice is considerably higher, rendering the fulfillment of the compositional and structural fluctuations required for the nucleation of the alumina phase easier. Therefore, the nucleation temperature of the alumina phase gradually decreases from 1500 °C at an Al_2_O_3_:SiO_2_ ratio of 1:1.5 to 1300 °C at an Al_2_O_3_:SiO_2_ ratio of 1:1.25 and eventually to 1100 °C at an Al_2_O_3_:SiO_2_ ratio of 1:1.

### 4.2. Analysis of the Catalytic Mechanism

Based on a previous study [16], when the Al_2_O_3_:SiO_2_ molar ratio was approximately equivalent to the atomic ratio of 3:2 mullite (3Al_2_O_3_·2SiO_2_), the topaz phase was initially generated during HF catalysis and subsequently transformed into mullite whiskers [6,7,8]. However, in this experiment, when the SiO_2_ and HF contents were simultaneously varied, the resulting intermediate products were different, reflecting the characteristics of two major catalytic reaction pathways. During F catalysis, mullite whiskers probably formed via two pathways, as described by reactions (1)–(7) [10,11,12,25].

Al_2_O_3_(s) + 6HF(g) → 2AlF_3_(g) + 3H_2_O(g), (1)

SiO_2_(s) + 4HF(g) → SiF_4_(g) + 2H_2_O(g), (2)

Pathway I

AlF_3_(g) + H_2_O(g) → AlOF(g) + 2HF(g), (3)

2AlOF(g) + SiF_4_(g) + 2H_2_O(g) → Al_2_SiO4F_2_(s) + 4HF(g), (4)

4Al_2_SiO_4_F_2_(s) → Al_6_Si_2_O_13_(s) + Al_2_O_3_(s) + 2SiF_4_(g), (5)

Pathway II

6AlF_3_(g) + 2SiF_4_(g) + 13H_2_O(g) → Al_6_Si_2_O_13_(s) + 26HF(g), (6)

4AlF_3_(s) + 3SiO_2_(s) → 2Al_2_O_3_(s) + 3SiF_4_(g), (7)

The phase analysis reveals that when the Al:Si ratio is approximately equivalent to the 3:2 stoichiometric ratio of the mullite phase, the F element catalyzes the reaction via pathway I, which involves reactions (1)–(5). F ions enter the Al–Si–O system via SiF_4_ to form the F-containing topaz phase (Al_8.0_Si_4.0_O_18.4_F_5.6_H_2.4_), which subsequently transforms into the mullite phase. The process is as follows.

Silicon-rich transient topaz phase (900 °C) → stable orthorhombic mullite (900–1500 °C) → recrystallization and precipitation of the alumina phase (1100–1500 °C).

The Al:Si ratio of the topaz phase is 2:1, and this phase has a higher silicon content than the 3:2-type mullite (3Al_2_O_3_·2SiO_2_). This type of lattice requires the reverse diffusion of Al atoms to form the mullite phase. Under this reaction mechanism, Al is preferentially involved in the low-temperature transformation of the topaz phase to the mullite phase, and consequently, numerous Al ions in the low-temperature topaz phase are consumed. Therefore, the formation of an aluminum-rich environment in the system is suppressed. Meanwhile, the phase transformations of mullite and alumina simultaneously occur, leading to a certain degree of competition. Although the alumina phase can nucleate at relatively low temperatures, the Al_2_O_3_ content at 1500 °C is comparatively low owing to the consumption of Al elements during the transformation of the mullite phase.

Conversely, when the SiO_2_ content is high and the Al_2_O_3_:SiO_2_ molar ratio is considerably lower than the 3:2 stoichiometric ratio of mullite, the aforementioned experimental results indicate that at ratios of 1:1.25 and 1:1.5, the system under F catalysis does not undergo reactions (4) and (5); instead, it follows pathway II, involving reactions (1), (2), (6), and (7) to form mullite whiskers. Meanwhile, the F element cannot enter the Al–Si–O system, and it directly reacts with AlF_3_ and SiF4 to form the mullite phase. In pathway II, the SiO_2_ and HF contents in the system are simultaneously reduced, leading to a reduction in the contents of SiF4 obtained via reaction (2) and the transient mullite phase formed via reaction (6), and eventually, the Al_2_O_3_ content increases.

In summary, the formation of mullite whiskers during HF catalysis is caused by the synergistic effects of the catalytic reaction on the phase transformation process in the Al–Si–O system. The A_l2_O_3_:SiO_2_ ratio influences the phase transformation process in the Al–Si–O system, and the HF catalysis interacts with this process. When the compositional ratios of the three components differ, the Al–Si–O system exhibits two distinct catalytic pathways and different phase transformation characteristics, which provides a reasonable explanation for the experimental results presented in Section 3.

## 5. Conclusions

(1) Single-crystal mullite whiskers were prepared via HF catalysis, and the growth direction of the whiskers is along the [001] crystallographic axis, as revealed via TEM and SAED analyses. Under the conditions of sufficient HF catalysis, the obtained product is entirely composed of mullite whiskers. When the Al_2_O_3_:HF ratio is maintained constant, a reduction in the SiO_2_ content leads to the formation of numerous flake-shaped structures in the product, as evidenced by the SEM images. The corresponding EDS analysis reveals the generation of Al_2_O_3_. A further reduction in the SiO_2_ content results in the complete transformation of the product into flake-shaped Al_2_O_3_, the diameter and thickness of which increase with decreasing SiO_2_ content. The effect of the reduction in the SiO_2_ content is analogous to that of an increase in the catalyst content, which is ascribed to the excessive consumption of Si in the system by fluorine during whisker growth. Therefore, the reduction in the SiO_2_ content under fully catalyzed conditions leads to the formation of an aluminum-rich system, generating numerous flake-shaped Al_2_O_3_ structures.
(2) SiO_2_ and HF exhibit synergistic effects on the growth of mullite whiskers during fluorine catalysis. When the SiO_2_ and HF contents in the system are simultaneously reduced, the resulting product comprises a mixture of mullite whiskers and flake-shaped alumina structures, with the latter component being dominant. However, a further reduction in both components leads to an increase in the content of mullite whiskers. The phase transformation in the system featuring different compositional ratios at 900–1500 °C indicates that: ① when the SiO_2_ and HF contents are relatively high (Al_2_O_3_:SiO_2_:HF = 1:1.5:4.2), the alumina phase begins to form at 1500 °C; however, when the contents of both components are reduced, the onset temperature for the formation of the alumina phase gradually decreases to 1100 °C. ② Different proportions of Al_2_O_3_, SiO_2_, and HF can lead to distinct catalytic processes. Specifically, at the lowest SiO_2_ and HF contents (Al_2_O_3_:SiO_2_:HF = 1:1:2.8), Si and F ions preferentially generate the silicon-rich topaz phase (Al_8.0_Si_4.0_O_18.4_F_5.6_H_2.4_), which gradually transforms into the mullite phase. Conversely, when the molar amount of SiO_2_ exceeds that of Al_2_O_3_, the aluminum-rich mullite phase is directly generated, precluding the formation of the topaz phase.
(3) The formation mechanism of mullite whiskers can be explained using the phase transformation in the Al–Si–O system, reasonably describing the experimental phenomena. Literature reports suggest that the mullite phase exhibits a lower activation energy for crystallization and a higher activation energy for growth, whereas the alumina phase demonstrates an opposite trend. Accordingly, during the phase transformation occurring in the Al–Si–O system, the transient alumina-rich mullite phase is first generated at low temperatures and gradually transforms into a stable orthorhombic mullite phase, which is followed by the recrystallization and precipitation of the alumina phase at high temperatures. During this process, the relatively low SiO_2_ content facilitates the formation of the alumina-rich mullite phase, which probably fulfills the compositional and structural fluctuations of alumina nucleation. Therefore, with the reduction in the SiO_2_ content, the formation temperature of the alumina phase decreases.
(4) Combining the literature findings and the experimental data obtained in this study, different ratios of Al_2_O_3_, SiO_2_, and HF lead to two distinct catalytic pathways. When the SiO_2_ and HF contents are relatively low and the Al:Si ratio is approximately equivalent to the stoichiometric ratio 3:2 of mullite, pathway I reactions occur. The F ions enter the Al–Si–O system via SiF_4_ to form the fluorine-containing topaz phase. However, when the SiO_2_ and HF contents are relatively high, pathway II reactions occur, and the mullite phase is directly generated via AlF_3_ and SiF_4_, precluding the formation of fluorine oxides. Notably, the initial formation of the topaz phase at relatively low temperatures consumes numerous Al ions, suppressing the formation of alumina at high temperatures. In pathway II, the simultaneous reduction in the SiO_2_ and HF contents leads to a decrease in the SiF_4_ content. Therefore, the content of the mullite phase decreases, which provides a plausible explanation for the phenomenon in which the group featuring an intermediate compositional ratio exhibits a lower content of mullite whiskers than the base group.

## Figures and Tables

**Table 1:** Experimental groups employed in this study.

**Influence of Si/Al Ratio**		
**Group**	**Molar ratio of Al_2_O_3_:SiO_2_:HF**	**Notes**
1	1:1.5:4.2	Base group
2	1:1.25:4.2	Fixed ratio of Al_2_O_3_ and HF
3	1:1:4.2	
**Synergistic influences of Si/Al ratio and catalyst content**		
**Group**	**Molar ratio of Al_2_O_3_:SiO_2_:HF**	**Notes**
1	1:1.5:4.2	Base group
2	1:1.25:3.5	Fixed ratio of SiO_2_/HF
3	1:1:2.8	

**Table 2:** Statistical analysis results for the morphological parameters of the mullite whiskers and flake-shaped material in Figure 2.

Sample No.	Al_2_O_3_:SiO_2_	Morphology	Diameter of Whisker/μm	Aspect Ratio	Diameter of Flake/μm	Ratio of Diameter/Thickness
2-a	1:1.5	Rod-shaped	1.6 ± 0.24	10.4 ± 1.87		
2-b	1:1.25	Flake-shaped + a few rod-shaped	2.1 ± 0.49	9.3 ± 2.79	38.7 ± 11.22	12.1 ± 1.45
2-c	1:1	Flake-shaped			45.5 ± 10.46	7.7 ± 1.00

Note: Statistical results are based on a sample size of N > 100 for each group. Data are presented as mean ± standard deviation.

**Table 3:** Phase composition and semiquantitative composition of whiskers featuring different Al_2_O_3_:SiO_2_:HF ratios.

Al_2_O_3_:SiO_2_:HF	1:1.5:4.2	1:1.25:3.5	1:1:2.8
Crystalline phase	M + A	M + A	M + A
Semiquantitative analysis/%	88 + 12	43 + 57	68 + 32

Note: M: Mullite Al_4.8_Si_1.2_O_9.6_ (96-900-1323), A: Aluminum Oxide Al_2_O_3_ (01-088-0826).

**Table 4:** Phase transformation and semiquantitative analyses of the mullite whiskers at different temperatures.

Temperature/°C	900	1100	1300	1500
1:1.5:4.2	M	M	M	M (88%) + A (12%)
1:1.25:3.5	M’ (72%) + M (28%)	M	M (80%) + A (20%)	M (43%) + A (57%)
1:1:2.8	T (35%) + M (65%)	M (77%) + A (23%)	M (72%) + A (28%)	M (68%) + A (32%)

Note: T: Topaz Al_8.0_Si_4.0_O_18.4_F_5.6_H_2.4_ (96-900-5628), M’: Mullite Al_5.6_5Si_0.35_O_9.175_ (01-082-1237), M: Mullite Al_4.8_Si_1.2_O_9.6_ (96-900-1323), A: Aluminum Oxide Al_2_O_3_ (01-088-0826).

## Data Availability

The original contributions presented in this study are included in the article. Further inquiries can be directed to the corresponding author.
